# Genetic Diversity of *Mycobacterium tuberculosis* from Guadalajara, Mexico and Identification of a Rare Multidrug Resistant Beijing Genotype

**DOI:** 10.1371/journal.pone.0118095

**Published:** 2015-02-19

**Authors:** Samantha Flores-Treviño, Rayo Morfín-Otero, Eduardo Rodríguez-Noriega, Esteban González-Díaz, Héctor R. Pérez-Gómez, Virgilio Bocanegra-García, Lucio Vera-Cabrera, Elvira Garza-González

**Affiliations:** 1 Servicio de Gastroenterología, Hospital Universitario Dr. José Eleuterio González, Universidad Autónoma de Nuevo León, Monterrey, Nuevo León, México; 2 Hospital Civil de Guadalajara, Fray Antonio Alcalde, y el Instituto de Patología Infecciosa y Experimental, Centro Universitario de Ciencias de la Salud, Universidad de Guadalajara, Guadalajara, Jalisco, México; 3 Laboratorio de Medicina de Conservación, Centro de Biotecnología Genómica, Instituto Politécnico Nacional, Reynosa, Tamaulipas, México; 4 Servicio de Dermatología, Hospital Universitario Dr. José Eleuterio González, Universidad Autónoma de Nuevo León, Monterrey, Nuevo León, México; 5 Departamento de Patología Clínica, Hospital Universitario Dr. José Eleuterio González, Universidad Autónoma de Nuevo León, Monterrey, Nuevo León, México; University of Padova, Medical School, ITALY

## Abstract

Determining the genetic diversity of *M. tuberculosis* strains allows identification of the distinct *Mycobacterium tuberculosis* genotypes responsible for tuberculosis in different regions. Several studies have reported the genetic diversity of *M. tuberculosis* strains in Mexico, but little information is available from the state of Jalisco. Therefore, the aim of this study was to determine the genetic diversity of *Mycobacterium tuberculosis* clinical isolates from Western Mexico. Sixty-eight *M. tuberculosis* isolates were tested for susceptibility to first-line drugs using manual Mycobacteria Growth Indicator Tube method and genotyped using spoligotyping and IS*6110*-restriction fragment length polymorphism (RFLP) pattern analyses. Forty-seven (69.1%) isolates were grouped into 10 clusters and 21 isolates displayed single patterns by spoligotyping. Three of the 21 single patterns corresponded to orphan patterns in the SITVITWEB database, and 1 new type that contained 2 isolates was created. The most prevalent lineages were T (38.2%), Haarlem (17.7%), LAM (17.7%), X (7.4%), S (5.9%), EAI (1.5%) and Beijing (1.5%). Six (12.8%) of the clustered isolates were MDR, and type 406 of the Beijing family was among the MDR isolates. Seventeen (26.2%) isolates were grouped into 8 clusters and 48 isolates displayed single patterns by IS*6110*-RFLP. Combination of IS*6110*-RFLP and spoligotyping reduced the clustering rate to 20.0%. The results show that T, Haarlem, and LAM are predominant lineages among clinical isolates of *M. tuberculosis* in Guadalajara, Mexico. Clustering rates indicated low transmission of MDR strains. We detected a rare Beijing genotype, SIT406, which was a highly resistant strain. This is the first report of this Beijing genotype in Latin America.

## Introduction

It is estimated that a third of the world’s population is infected with *Mycobacterium tuberculosis* and over 9 million new tuberculosis (TB) cases are diagnosed annually [1]. In Mexico, more than 2000 people die every year from TB, and the rate is 16.8 cases per 100,000 in 2010. In the western state of Jalisco, the TB rate was 13 per 100,000 [2]. Multidrug-resistant strains of *M*. *tuberculosis* (MDR-TB) resistant to isoniazid (INH) and rifampicin (RIF) are a challenge for clinicians. Patients with MDR-TB are significantly more difficult to treat and mortality is higher than in drug-susceptible cases [3].

Genotyping has been used to study TB transmission [4,5]. One genotyping technique is restriction fragment length polymorphism (RFLP) analysis, where the distribution and number of copies of an insertion sequence, IS*6110*, in a chromosome is monitored, and this event varies among different strains. Spoligotyping is based on polymorphisms in the direct repeat (DR) locus of the *M*. *tuberculosis* genome. Identification of identical genotypes from different patients generally indicates recent infection by a common source [6], although this greatly depends on the genotyping method used. Spoligotyping has gained international approval as a robust, fast, and reproducible typing method suitable for epidemiological research [7].

Several studies have reported the genetic diversity of *M*. *tuberculosis* strains in Mexico, but little information is available from the state of Jalisco [8–17]. Previously we evaluated phenotypic and genotypic resistance of *M*. *tuberculosis* isolates from Guadalajara, Jalisco [18]. Therefore, the aim of this study was to analyze the genetic diversity of *M*. *tuberculosis* clinical isolates from patients with pulmonary TB in Guadalajara, Jalisco. Shared types, clade predominance, and clustering rates of both susceptible and MDR isolates were determined.

## Methods

### Ethics Statement

The local ethics committee (Comité de Ética y Comité de Investigación, Facultad de Medicina y Hospital “Dr José Eleuterio González”, Universidad Autónoma de Nuevo León, Mexico) approved the study and written informed consent to participate in this study was obtained from each patient.

### Clinical specimens and isolates

The Hospital Civil de Guadalajara, Fray Antonio Alcalde, admitted more than 240 patients with TB between 2005 and 2011, mostly patients from Jalisco and the surrounding Western states. From September 2010 to November 2011, specimens (*n* = 351) were referred to the collaborative mycobacteriology laboratory for further testing. All samples were decontaminated by the Petroff method [19] and cultured on Löwestein-Jensen slants at 37°C. Smears were made and stained using the Ziehl-Neelsen method and were examined for the presence of acid-fast bacilli. The cultures were examined twice a week and their growth rates and colony morphologies were recorded. Isolates were identified by traditional biochemical methods including niacin production and nitrate reduction tests. One isolate per patient was assayed.

### DNA isolation and species identification

Mycobacteria were first subjected to enzymatic lysis with lysozyme and proteinase K. DNA was then obtained using a phenol extraction and ethanol precipitation [20]. Species identification was performed using multiplex PCR amplification of the *cfp32* (Rv0577), RD9 (Rv2073c) and RD12 (Rv3120) genes [21,22].

### Drug susceptibility testing

Drug susceptibilities were tested in all *M*. *tuberculosis* isolates using the manual Mycobacteria Growth Indicator Tube (MGIT) method. Critical concentrations of STR (0.8 μg/mL), INH (0.1 μg/mL), RIF (1.0 μg/mL), and EMB (3.5 μg/mL) were used according to the manufacturer’s instructions (Becton Dickinson and Co., Sparks Glencoe, MD) [23]. Fluorescence at 365_nm_ indicating microbial growth was detected with a UV light from a transilluminator. The susceptible H37Ra strain was used as a control.

### Spoligotyping analysis

Spoligotyping was performed as described previously [5] using a commercially available kit (Isogen Life Science, De Meern, Netherlands) according to the manufacturer’s instructions. Spoligotype results were compared to the SITVITWEB database (available at http://www.pasteur-guadeloupe.fr:8081/SITVIT_ONLINE/). At the time of the study, SITVITWEB contained 62,582 clinical isolates with 153 countries of origin [7]. A dendrogram of spoligotype patterns was generated with the unweighted pair group method and the neighbor joining algorithm using the online MIRU-VTRN *plus* application [24].

### IS*6110*-RFLP analysis

IS*6110*-RFLP analysis was performed according to the international standard method [4]. Comparison of the results was done using the IBM SPSS statistical software for Windows version 20.0 (SPSS Inc, Chicago, IL, USA). Isolates with clustered IS*6110*-RFLP banding patterns containing more than five bands were considered related. A dendrogram was generated with the neighbor joining algorithm using the Jaccard coefficient.

## Results

### Patients and strains

Clinical samples from 351 patients with pulmonary TB were collected and processed. From these, 68 *M*. *tuberculosis* isolates were obtained. Patients were predominantly male (53/68, 77.9%). The median age was 43 years (ranging 1 to 85 years).

### Shared types, clades, and clusters of *M*. *tuberculosis* isolates

From the 68 isolates, 31 different spoligotype patterns were detected. Unique spoligotype patterns were detected in 3 strains. They did not match any preexisting patterns in the SITVITWEB database and were considered orphans (Table 1). A total of 27 SITs (including 63 isolates) matched pre-existing SITs in the SITVITWEB database, and one new SIT containing 2 isolates was created in this study (Table 2).

**Table 1:** Description of orphan strains found in this study.

Strain ID	Sex	Age	Spoligotype Description	Octal Code	Drug Resistance
MEX47	Male	26	□□□□□□□□□□□□■■■■■□□■■■■□□□□□□□□□□□□□□□□■■■■	000076360000071	Susceptible
MEX224	Female	38	■■■■■■■■□□■■■□■■■■■■■■■■■■■■■■■■□□□□■□■■■■■	776357777760571	RIF Resistant
MEX257	Male	56	■■■■■■□□□□□□□■■■■■■■■■■■■■■■■■■■□□□□■■■■■■■	770037777760771	Susceptible

Orphan strains had spoligotype patterns that did not match a preexisting pattern in the SITVITWEB database.

**Table 2:** Description of shared types found in this study.

SIT^*a*^	Spoligotype Description	Octal Code	Clade^*b*^	*n* (%)
2	□□□□□□□□□□□□□□□□□□□□□□□□■□□□□□□■□□□□■■■■■■■	000000004020771	H2	1 (1.5)
17	■■□■■■■■■■■■□■■■■■■■□□□□■■■■■■■■□□□□■■■■■■■	677737607760771	LAM2	4 (5.9)
19	■■□■■■■■■■■■■■■■■■■□□■■■■■■■□□□□■□■■■■■■■■■	677777477413771	EAI2-Manila	1 (1.5)
20	■■□■■■■■■■■■■■■■■■■■□□□□■■■■■■■■□□□□■■■■■■■	677777607760771	LAM1	1 (1.5)
34	■■■■■■■■□□□□■■■■■■■■■■■■■■■■■■■■□□□□■■■■■■■	776377777760771	S	2 (2.9)
42	■■■■■■■■■■■■■■■■■■■■□□□□■■■■■■■■□□□□■■■■■■■	777777607760771	LAM9	5 (7.4)
47	■■■■■■■■■■■■■■■■■■■■■■■■□□□□□□□■□□□□■■■■■■■	777777774020771	H1	2 (2.9)
50	■■■■■■■■■■■■■■■■■■■■■■■■■■■■■■□■□□□□■■■■■■■	777777777720771	H3	6 (8.8)
51	■■■■■■■■■■■■■■■■■■■■■■■■■■■■■■■■□□□□■■■□□□□	777777777760700	T1	2 (2.9)
53	■■■■■■■■■■■■■■■■■■■■■■■■■■■■■■■■□□□□■■■■■■■	777777777760771	T1	17 (25)
92	■■■□□□□□□□□□■■■■■□■■■■■■■■■■■■■■□□□□■■■■■■■	700076777760771	X3	1 (1.5)
137	■■■■■■■■■■■■■■■■■□■■■■■■■■■■■■■■□□□□■■□□□□■	777776777760601	X2	3 (4.4)
232	■■■■■■■■■■■■■■■■■■■■■■□□□□□□□□□□□□□□□■■■■■■	777777760000171	Unk	1 (1.5)
335	■■■■■■■■■■■■■■■■■■■■□□□□■■■■■■□■□□□□■■■■■■■	777777607720771	Unk	1 (1.5)
371	■■■■■■■■■■■■■■■■■■□□□□□□■■■■■■□■□□□□■■■■■■■	777777007720771	H3	1 (1.5)
406	□□□□□□□□□□□□□□□□□□□□□□□□□□□□□□□□□□□□■■■□■■■	000000000000731	Beijing	1 (1.5)
469	□□□■■■■■■■■■■■■■■■■■□□□□■■■■■■■■□□□□■■■■■■■	077777607760771	LAM9	1 (1.5)
478	■■□□□■■■■■■■■■■■■□■■■■■■■■■■■■■■□□□□■■□□□□■	617776777760601	X2	1 (1.5)
535	■■■■■■■■■■■■■■■■■■■■■□□□■■■■■■■■□□□□■■■■■■■	777777707760771	T1	1 (1.5)
732	■■■■■■■■■■■■■■□□■■■■■■■■■■■■■■■■□□□□■■■■■■■	777763777760771	T1	1 (1.5)
1215	■■□■■■■■■■■■□■■■□■■■□□□□■■■■■■■■□□□□■■■■■■■	677735607760771	LAM2	1 (1.5)
1225	■■■■■■■■□□■■■■■■■■■■■□□□□□□□□■■■□□□□■■■■■■■	776377700160771	S	1 (1.5)
1231	■■■■■■■■■■■■■■■■□□□□□■□■■■■■■■■■□□□□■■■■■■■	777774057760771	T5	4 (5.9)
1356	■■■■■■■■□□■■■■■■■■■■■■■■■■■■■■■■□□□□■■■■□■■	776377777760751	S	1 (1.5)
1686	■■■■■■■■■■■■■□□□□□□□□□□□■□□□□□□■□□□□■■■■■■■	777740004020771	H1	1 (1.5)
2007	■■■■■■■■■■■■□■■■■■■■□■■■■■■■■■■■□□□□■■■■■■■	777737677760771	T3	1 (1.5)
2212	■■■■■■■■■■■■■■■■■■■□□□□□■□□■■■□■□□□□■□■■■■■	777777404720571	H3	1 (1.5)
3434*	■■■■■■■□□■■■■□□□□□□□□□□■□■■■■□■□□□□□■■■■■■■	774740005720771	Unk	2 (2.9)

^*a*^In the SITVITWEB database, the spoligo (shared) international type (SIT) numbers designate spoligotypes shared by two or more patient isolates.

*SIT3434 is a newly created shared type due to two strains belonging to a new pattern within this study.

^*b*^Clade designations according to the SITVITWEB database: Beijing clade, East African-Indian (EAI) clade and 9 sublineages, Haarlem (H) clade and 3 sublineages, Latin American-Mediterranean (LAM) clade and 12 sublineages, the ancestral “Manu” family and 3 sublineages, the S clade, the IS*6110*-low-banding X clade and 3 sublineages, and an ill-defined T clade with 5 sublineages. Unk, unknown patterns within any of the major clades described in the database.

**Fig 1:**
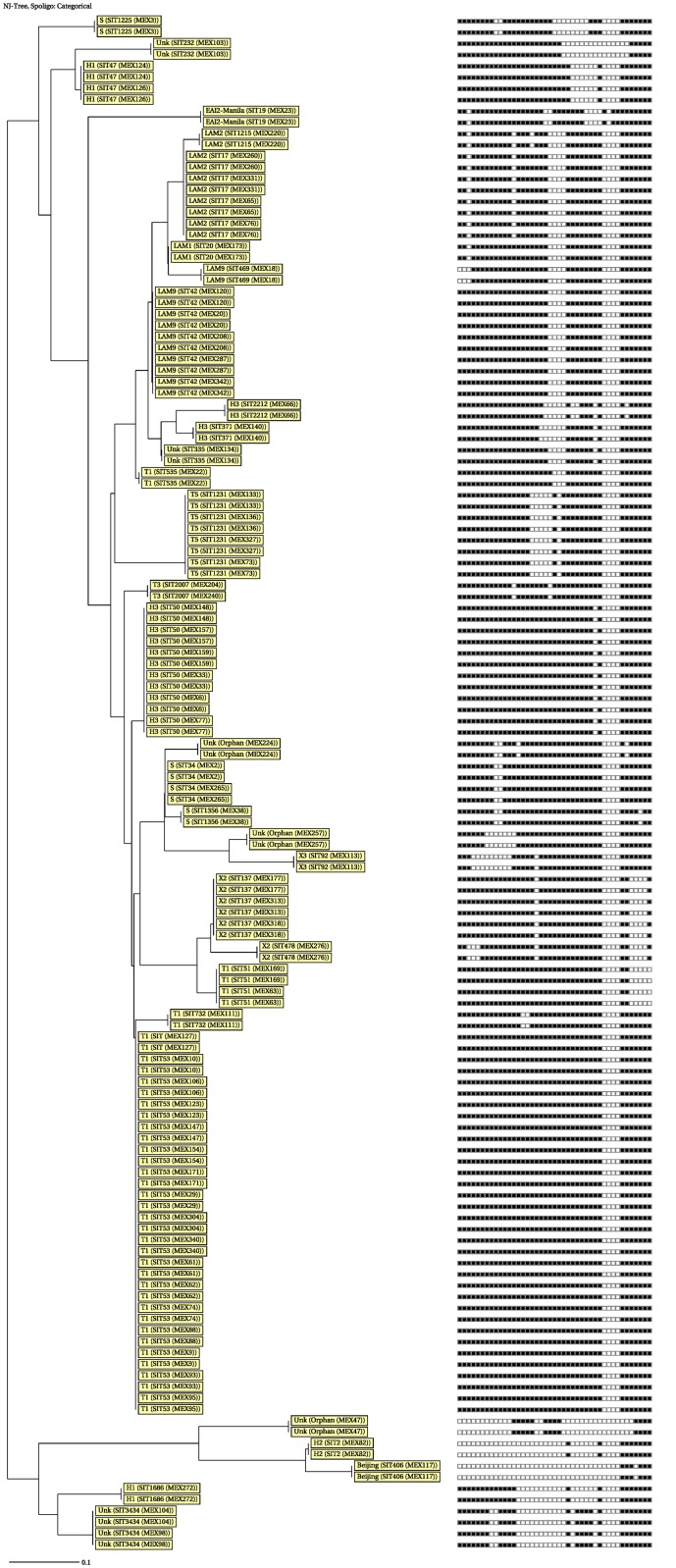
Spoligotype tree of *M. tuberculosis* isolates (n = 68). Lineage, Spoligotype International Type (SIT) as defined in the SITVITWEB proprietary database of the Pasteur Institute of Guadeloupe, and the strain identifications are shown.

Clustering was detected in 47 isolates (69.1%) (Fig. 1) including SIT53 (T1 of ill-defined T clade, *n* = 17, 25.0%), SIT50 (H3 of Haarlem clade, *n* = 6, 8.8%), SIT42 (LAM9 of the Latin American-Mediterranean clade, *n* = 5, 7.4%), SIT17 (LAM2, *n* = 4, 5.9%), SIT1231 (T5, *n* = 4, 5.9%), SIT137 (X2 of the IS*6110*-low-banding X clade, *n* = 3, 4.4%), SIT34 (the S clade, *n* = 2, 2.9%), SIT47 (H1, *n* = 2, 2.9%), SIT51 (T1, *n* = 2, 2.9%), and SIT3434 (Unknown clade, n = 2, 2.9%), which was created in this study. Single patterns were observed in 18 isolates including SIT2 (H2), SIT19 (EAI2-Manila), SIT20 (LAM1), SIT92 (X3), SIT232 (Unk), SIT335 (Unk), SIT371 (H3), SIT406 (Beijing), SIT469 (LAM9), SIT478 (X2), SIT535 (T1), SIT732 (T1), SIT1215 (LAM2), SIT1225 (S), SIT1356 (S), SIT1686 (H1), SIT2007 (T3) and SIT2212 (H3).

### IS*6110*-RFLP patterns of *M*. *tuberculosis* isolates

**Fig 2:**
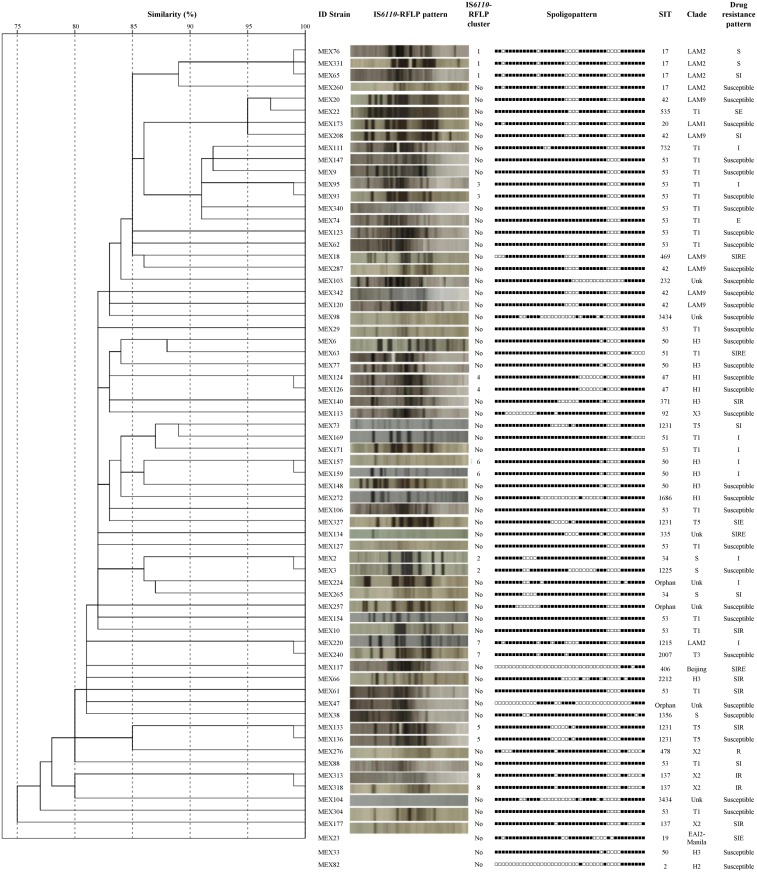
IS6110-RFLP clustering patterns, spoligopatterns and drug resistance profiles of *M. tuberculosis* isolates. Drug resistance profile indicates resistance to I: isoniazid; R: rifampicin; S: streptomycin and E: ethambutol.

IS*6110*-RFLP patterns of three isolates (MEX23, MEX33 and MEX82) were not obtained despite three attempts. The remaining 65 isolates were typified by IS*6110*-RFLP typing (Fig. 2). We identified 56 fingerprint patterns, 8 were clustered including 17 isolates with a clustering rate of 26.2% and the remaining 48 had single patterns. The number of IS*6110* copies varied from 1 to 14; 13.8% (*n* = 9) of the isolates were low-copy-number isolates having less than six copies of IS*6110*. Combination of IS*6110*-RFLP and spoligotyping reduced the number of clustered isolates to 13 and the clustering rate to 20.0%. Isolates lacking the IS*6110* element were not observed.

### Drug resistant *M*. *tuberculosis* isolates

Drug resistance was detected in 33 (48.5%) isolates. Among the 13 MDR isolates, SIT53 (*n* = 3) and SIT137 (*n* = 3) were clustered by spoligotyping and only 2 isolates (MEX313 and MEX318) were clustered by IS*6110*-RFLP (Fig. 2). The single pattern isolates SIT335 (Unk), SIT371 (H3), SIT406 (Beijing), SIT469 (LAM9) and SIT2212 (H3) were also MDR.

## Discussion

Determining the genetic diversity of *M*. *tuberculosis* strains allows identification of the distinct *M*. *tuberculosis* genotypes responsible for TB in different regions. Genetic variation in *M*. *tuberculosis* strains contributes to the variability in disease presentation, frequency of transmission, and treatment outcomes [25,26]. Knowing these factors is important for appropriate treatment and control of TB.

The most predominant clades found in our study were the ill-defined T clade (SIT53), the Haarlem clade (SIT50), the Latin American-Mediterranean clade (SIT42) and the IS*6110*-low-banding X clade (SIT137). These results are similar to those reported in several studies from across the country [8–17]. And considering that SIT42 evolved originally from SIT53, this group is very dominant in this region [27].

We detected high clustering rates of spoligotypes (69.1%) consistent with previous reports in Mexico [9,11–13]. However, despite the single spoligopattern presented, four isolates (MEX2, MEX3, MEX220 and MEX240) were in 2 clusters according to IS*6110*-RFLP. Thus, combination of IS*6110*-RFLP and spoligotyping reduced the clustering rate to 20.0%. This indicates that spoligotyping alone is not discriminatory enough to determine epidemiological links and for transmission analysis, due to the high rate of homoplasy (independent mutational events that result in the loss of the same spacer) exhibited by the spacers used in spoligotyping [28–30].

The low clustering rate detected in this study indicates low transmission of *M*. *tuberculosis* clones. Most of the clustered isolates, regardless of genotypic family, were drug susceptible, also indicating low transmission of MDR isolates. In addition, changes in the drug resistance pattern of the isolates in one cluster were not accompanied by changes of the IS*6110*-RFLP pattern (clusters 1, 2, 3, 5 and 7). This is in accordance with previous studies where the rate of change of IS6110 fingerprint patterns in drug-resistant isolates seems not to differ from that in drug-susceptible isolates, thus indicating a high degree of stability of IS6110 patterns [31–33].

Although a high number of previously reported spoligotypes were identified in Mexico [12,13,17], 19.1% of our isolates showed SIT (335, 371, 406, 469, 1231, 1686, 2007, 2212 and 3434) which were previously unreported in the country.

*M*. *tuberculosis* isolates with no IS*6110* elements are rare [34] and we did not find them in Jalisco. In Mexico, there is only one report in Mexico City, constituting as high as 20% of the isolates studied [13]. It will be important to determine the ancestry of these patients, since zero IS*6110 M*. *tuberculosis* isolates have been found elsewhere in Mexico.

Some *M*. *tuberculosis* strains of the Beijing genotype have been often associated with MDR phenotype and higher transmission compared to other genotypes [26,35]. The Beijing genotype is principally identified by the deletion of spacers 1–34 and the presence of at least 3 of the 9 spacers between positions 35 and 43 of the DR locus [36–38]. To date, 27 Beijing family genotypes have been reported in the STVIT2 international database (including 6,148 strains from 71 countries). SIT1 was the classical Beijing genotype in the SITVITWEB database at the time this study was conducted. Although these strains are prevalent throughout Asia, they are found worldwide [39]. The SIT1 genotype has been reported in several states in Mexico including Puebla [14], Jalisco [9], Baja California, Sinaloa, Veracruz [15] and San Luis Potosi [16]. Both drug-susceptible and resistant strains have been documented.

In this study, we detected an MDR strain with the Beijing genotype and a SIT406 pattern. To our knowledge, this is the first report of Beijing SIT406 in Mexico or Latin America. At the time of this comparison, SIT406 represented a total of 14 strains in the SITVITWEB database, which corresponded to 0.2% (14/6,148) of all Beijing strains, in contrast to SIT1, which corresponded to 94.3% (5,800/6,148) of all Beijing strains. According to the available entries from the database, the distribution of SIT406 strains is limited to the United States (64.3%), Belgium, India, Italy, Japan, and Tunisia (7.1% each).

Little is known about the differences between disease caused by the rare Beijing and classical Beijing genotypes. In Latin America only two rare Beijing strains have been reported in Mexico (SIT269 and SIT941) [7] and one case was reported in Colombia (SIT190) [40]. TB treatment outcomes are associated with MDR phenotypes rather than Beijing genotypes [41]. However, most studies of Beijing strains only include SIT1 strains, and the behavior of other Beijing family genotypes is undefined. Spoligotyping is commonly used in high-income countries and data from countries such as Mexico is limited.

One limitation of this work is the lack of use of mycobacterium interspersed repetitive unit-variable number of tandem repeat (MIRU-VNTR) as a genotyping method for *M*. *tuberculosis* isolates. It is well known that in the *M*. *tuberculosis* epidemiology, MIRU-VNTR and spoligotyping are increasingly being used as first line methods for their ease, discriminatory power, and digital analysis of generated data [6] compared with the gold standard IS*6110* typing method. Nonetheless, MIRU-VNTR genotyping is commonly used in high-income countries and availability in countries such as Mexico is limited. Incidentally, it has been reported a reduced discriminatory power of 24-loci MIRU-VNTR compared to IS*6110* typing especially in Beijing lineages [42,43]. In these cases, 24-loci MIRU-VNTR typing is inadequate for accurately identifying transmission chains of Beijing strains, and thus needs to be complemented either by IS*6110* typing or combinations of MIRU-VNTR loci more variable in Beijing strains [43]. Contrariwise, MIRU-VNTR allows a higher discrimination of the low-copy-number isolates, which in our case corresponded to 13.8% of the isolates studied. Therefore, an expansion of this work is required to further analyze this isolates using MIRU-VNTR, both to allow the inclusion of a greater sample number and to further describe the Beijing genotype detected in this study.

In summary, T, Haarlem, and LAM were predominant lineages in clinical isolates of *M*. *tuberculosis* in Guadalajara, Jalisco. Transmission of MDR strains was low in Guadalajara. However, some isolated cases of TB were caused by the rare MDR Beijing genotype. The report of an MDR strain with a SIT406 pattern indicated additional effort should be placed on the search for these genotypes. The status of Beijing strains in Mexico needs to be monitored to determine whether they are expanding.
